# Neonatal Exposure to Bisphenol A Alters Reproductive Parameters and Gonadotropin Releasing Hormone Signaling in Female Rats

**DOI:** 10.1289/ehp.0800267

**Published:** 2009-01-07

**Authors:** Marina Fernández, Maria Bianchi, Victoria Lux-Lantos, Carlos Libertun

**Affiliations:** 1 Instituto de Biología y Medicina Experimental, Consejo Nacional de Investigaciones Científicas y Técnicas, Buenos Aires, Argentina;; 2 Facultad de Medicina, Universidad de Buenos Aires, Argentina

**Keywords:** Bisphenol A, estrous cycle, GnRH pulsatility, GnRH signaling, gonadotropins, puberty

## Abstract

**Background:**

Bisphenol A (BPA) is a component of polycarbonate plastics, epoxy resins, and polystyrene and is found in many products. Several reports have revealed potent *in vivo* effects, because BPA acts as an estrogen agonist and/or antagonist and as an androgen and thyroid hormone antagonist.

**Objectives:**

We analyzed the effects of neonatal exposure to BPA on the reproductive axis of female Sprague-Dawley rats.

**Methods:**

Female rats were injected subcutaneusly, daily, from postnatal day 1 (PND1) to PND10 with BPA [500 μg/50 μL (high) or 50 μg/50 μL (low)] in castor oil or with castor oil vehicle alone. We studied body weight and age at vaginal opening, estrous cycles, and pituitary hormone release *in vivo* and *in vitro,* as well as gonadotropin-releasing hormone (GnRH) pulsatility at PND13 and in adults. We also analyzed two GnRH-activated signaling pathways in the adults: inositol-triphosphate (IP_3_), and extracellular signal-regulated kinase_1/2_ (ERK_1/2_).

**Results:**

Exposure to BPA altered pituitary function in infantile rats, lowering basal and GnRH-induced luteinizing hormone (LH) and increasing GnRH pulsatility. BPA dose-dependently accelerated puberty onset and altered estrous cyclicity, with the high dose causing permanent estrus. In adults treated neonatally with BPA, GnRH-induced LH secretion *in vivo* was decreased and GnRH pulsatility remained disrupted. *In vitro*, pituitary cells from animals treated with BPA showed lower basal LH and dose-dependently affected GnRH-induced IP_3_ formation; the high dose also impaired GnRH-induced LH secretion. Both doses altered ERK_1/2_ activation.

**Conclusions:**

Neonatal exposure to BPA altered reproductive parameters and hypothalamic–pituitary function in female rats. To our knowledge, these results demonstrate for the first time that neonatal *in vivo* BPA permanently affects GnRH pulsatility and pituitary GnRH signaling.

Humans and wildlife are exposed to a variety of contaminants, such as xenoestrogens, on a daily basis. Bisphenol A (BPA), a xenoestrogen, is a constituent of polycarbonate plastics and epoxy resins used in dentistry and in the food industry. The polymer bonds hydrolyze at high temperatures and release BPA. BPA could be ingested by humans; detectable amounts have been found in food cans, microwave containers, polycarbonate bottles, and in human saliva after treatment with dental sealants (Vandenberg et al. 2007). Although results of some *in vitro* assays have suggested that BPA is a weak environmental estrogen (Vandenberg et al. 2007; Wetherill et al. 2007), important *in vivo* effects have been found using a wide range of doses, animal models, and study end points. The current U.S. Environmental Protection Agency (EPA) reference dose [50 μg/kg/day (U.S. EPA 1993)] was calculated by dividing the lowest observed adverse effect level (LOAEL; 50 mg/kg/day) by 1,000. For the present study, we defined low doses as those < LOAEL, as reported by Richter et al. (2007). Effects of BPA can be influenced by species, strain, dose, and time of exposure. Adult animals exposed to BPA show effects that are reversible when the exposure ceases (Richter et al. 2007). In contrast, perinatal or neonatal exposure produces organizational effects that are irreversible in Sprague-Dawley (Kato et al. 2003; Moral et al. 2008; Patisaul et al. 2006; Rubin et al. 2001), Wistar (Durando et al. 2007; Ramos et al. 2003), and Fisher 344 rats (Khurana et al. 2000). These permanent effects are in agreement with the actions of estrogens during development, ranging from the establishment of sex differences to pervasive trophic and neuro protective effects (McCarthy 2008).

In addition to its estrogenicity, BPA can antagonize the effects of estrogens, androgens, or thyroid hormones; act through non-genomic pathways; and influence enzyme activity or receptor expression (Wetherill et al. 2007).

Gonadotropin-releasing hormone (GnRH) is a hypothalamic decapeptide critical for normal mammalian reproductive function. GnRH secretion is pulsatile and acts on gonadotropes to stimulate synthesis and release of luteinizing hormone (LH) and follicle-stimulating hormone (FSH) (Pawson et al. 2005). Gonadotropins are composed of an alpha subunit, common to both, and beta subunits, unique for each of them. The frequency of the GnRH pulses determines the predominant subunit transcribed at a given time. LHβ is transcribed at fast GnRH pulses, whereas FSHβ is favored at slow pulse frequencies (Ferris and Shupnik 2006).

After binding of GnRH to its receptor (a G_q/11_-coupled receptor), phospholipase C is activated, leading to diacylglycerol and inositol 1,4,5-triphosphate (IP_3_) formation (Naor et al. 1986). Subsequently, IP_3_ induces an increase of intracellular calcium (leading to gonadotropin release) and protein kinase C (PKC) is activated, resulting in multiple cellular responses, such as activation of the mitogen-activated protein kinase (MAPK) family (Sundaresan et al. 1996).

In this study we explored the effects of neonatal exposure to high and low doses of BPA on reproductive parameters in female Sprague-Dawley rats, a strain reported to have low sensitivity to estrogenic compounds (Steinmetz et al. 1997). We analyzed puberty onset, estrous cyclicity, *in vivo* and *in vitro* pituitary response to GnRH, GnRH pulsatility, and GnRH-activated signaling pathways.

## Materials and Methods

### Animals

Studies were performed according to protocols for animal use (Institute of Laboratory Animal Resources 1996) and approved by the institutional animal care and use committee of the Instituto de Biología y Medicina Experimental, Consejo Nacional de Investigaciones Científicas y Técnicas (IByME-CONICET). Animals were treated humanely and with regard for alleviation of suffering.

Sprague-Dawley rats (200–250 g) from the IByME colony, established from Charles River stock in 1985, were maintained under a controlled 12-hr light/dark cycle and temperature conditions. They were housed in steel cages with wood shavings as bedding material and given free access to commercial laboratory chow (Gepsa Feeds, Grupo Pilar S.A, Córdoba, Argentina) and tap water in glass bottles. We did not test the phytoestrogen concentration in the food, but we assumed that all animals were exposed to the same levels, since food intake was equivalent between groups.

Pregnant females were housed individually; on the day of birth [postnatal day (PND) 1], litters were reduced to eight pups. Female neonates from each litter were assigned to the different experimental groups and left with the dam. On PND1–PND10, each pup received a daily subcutaneous injection of BPA (Aldrich, Milwaukee, WI, USA) in castor oil [50 μg/50 μL (BPA50; dose range: 6.2–2.5 mg/kg body weight); 500 μg/50 μL (BPA500; 62.5–25.0 mg/kg)] or castor oil vehicle (control). BPA500 has been used previously in this and other strains of rats (Khurana et al. 2000; Patisaul et al. 2006, 2007); because it is slightly > LOAEL, we consider it to be a high dose. We consider 50 μg/50 μL BPA to be a low dose because it is below the LOAEL.

### First experimental design

On PND13, an age in which the pituitary is highly sensitive to stimulatory and inhibitory inputs (Becu-Villalobos et al. 1990), animals treated with vehicle (control), BPA50, or BPA500 on PND1–10 were killed and trunk blood was collected. We then determined serum prolactin (PRL) by radioimmunoassay (RIA).

### *In vivo* GnRH-induced gonadotropin release

On PND13, we injected 9–12 animals from each group intraperitoneally with 100 ng GnRH (Bachem, Torrance, CA, USA) or saline solution. After 15 min (LH) or 50 min (FSH), trunk blood was collected; serum LH and FSH were then determined by RIA.

### GnRH pulsatility

We performed pulsatility studies *ex vivo* as described by Heger et al. (2003). Briefly, On PND13, preoptic area-anterior hypothalamus–medial basal hypothalamus (POA-MBH) were collected and incubated in 1.5 mL microfuge tubes containing 250 μL Krebs-Ringer bicarbonate buffer with 4.5 mg/mL glucose and 16 mM HEPES at 37°C for 6 hr. After 30 min preincubation, the medium from each flask was renewed at 9-min intervals; medium was stored (−20°C) for GnRH measurement by RIA.

We identified GnRH pulses and defined their parameters using Cluster8 computer algorithm analysis developed by Veldhuis and Johnson (1986) and obtained from M.L. Johnson (University of Virginia; available: http://mljohnson.pharm.virginia.edu/home.html). We used a 2 × 2 cluster configuration and a *t* statistic of 2 for the upstroke and downstroke to maintain false-positive and false-negative error rates < 10%, as suggested by Martinez de la Escalera et al. (1992). The experiment was repeated four times, including one animal from each group per experiment.

### *In vitro* GnRH-induced gonadotropin release

Using high-dose animals and controls on PDN13, we obtained anterior pituitary cells as described by Mongiat et al. (2006); we used three pituitaries from each group for each culture. Cells were plated (50,000 cells/well) in complete medium; after 5 days, they were washed with serum-free medium (SFM) and stimulated with GnRH 1.10^−9^ M and 1.10^−7^ M (1 hr). Media were stored (−20°C) for gonadotropin analysis by RIA. The experiment was performed in quadruplicate (*n* = 7 per treatment group).

### Second experimental design

After weaning on PND21, 11–15 pups from each treatment group (control, BPA50, and BPA500) were weighed daily and the age and weight at vaginal opening (VO) were recorded. Animals were multiple-housed (four per cage), keeping members of each litter together. We determined estrous cycles in five animals from each group from PND60 to PND120 by examining vaginal smears under a light microscope. After PND120, serum hormones, pituitary response to GnRH, and GnRH pulsatility were determined in animals in estrus.

### *In vivo* GnRH-induced gonadotropin release

For this experiment we used seven adult animals from each treatment group. Under ketamine/xylazine anesthesia (60/10 mg/kg body weight), one blood sample was collected from the jugular vein (0 min) and GnRH (100 ng) was administered into the same vein. Further samples were taken at 15 and 50 min. Serum LH was determined by RIA.

### GnRH pulsatility

Experiments and analysis were performed as described above (*n* = 7).

### Inositol phosphate determination

Levels of inositol monophosphate (IP_1_), biphosphate (IP_2_), and IP_3_ were measured as described by Mongiat et al. (2004). Pituitary cells from adult animals in each treatment group were plated (500,000 cells/well) in complete medium, with two pituitaries from each experimental group used per culture. After 4 days, cells were incubated for 48 hr with 4 μCi/mL/well of [2-^3^H(N)]-myo-inositol (PerkinElmer Life Sciences, Wallac Oy, Turku, Finland), washed with SFM, and incubated for 15 min in the presence of 20 mM lithium chloride. Stimuli were added [final concentrations: GnRH, 1.10^−7^ M; GnRH-antagonist Cetrorelix (CRX), 1.10^−6^ M (Serono, Buenos Aires, Argentina)], and cells were incubated for 30 min. After incubation, media were collected to measure LH, and cells were scraped in 0.5% perchloric acid; lysates were then transferred to microfuge tubes, neutralized with 0.72 M potassium hydroxide and 0.6 M potassium bicarbonate, and centrifuged for 10 min at 3,000 rpm. Supernatants were chromatographed in ionic exchange columns (Dowex AG1-XP Resin, 200–400 mesh, formate form; Bio-Rad Laboratories, Hercules, CA, USA) to elute free inositol, IP_1_, IP_2_, and IP_3_. Aliquots of the eluates were mixed with OptiPhase “Hisafe” 3 (PerkinElmer) and counted in a liquid scintillation counter; results are expressed as IP_3_/inositol (*n* = 6 per treatment group).

### GnRH-induced activation of extra cellular signal-regulated kinase_1/2_ (ERK_1/2_)

We determined ERK_1/2_ activation by Western blot analysis (Chamson-Reig et al. 2003). Pituitary cells from adult animals were plated (250,000 cells/well) as described previously, using one pituitary from each treatment group per culture. After 5 days, cells were washed with SFM and stimulated for 0, 5, 15, or 30 min with GnRH (1.10^−7^ M). Media were then removed and sample buffer (80 μL) added to the wells; plates were stored at −70°C. Cell lysates (20 μL) were run on 12% SDS-PAGE gels (Bio-Rad) and electro transferred to nitro-cellulose membranes, which were then blocked with 5% BSA–0.1% Tween-phosphate buffered saline (TPBS) for 2 hr at room temperature (RT). Membranes were incubated with a mouse phospho-specific ERK_1/2_ antibody (pERK_1/2_, sc-7383, 1:1,500; Santa Cruz Biotechnology) for 2 hr at RT. Membranes were stripped, blocked with 3% fat-free milk–TPBS buffer (1 hr at RT), and probed for 1 hr at RT with a rabbit polyclonal antibody that detects total ERK_1/2_ (sc-94, 1:1200; Santa Cruz Biotechnology). Signals were detected with horseradish peroxidase-conjugated anti-mouse antibody (1:2000, 1 hr at RT) or anti-rabbit antibody (1:4000, 2 hr at RT) and visualized by chemiluminescence. Immunoblots were quantified using Scion Image Software (Scion Corporation, Frederick, MD, USA); results are expressed as pERK_1/2_/total ERK_1/2_ (*n* = 4 per treatment group).

### Hormone dosage

We determined hormones by RIA using kits obtained from the National Hormone and Peptide Program (Torrance, CA, USA). Results are expressed in terms of reference preparation 3 rat LH, FSH, and PRL standards. Assay sensitivities were 0.015 ng/mL for LH, 0.1175 ng/mL for FSH, and 0.04 ng/mL for PRL. Intraassay and interassay coefficients of variation, respectively, were as follows: LH, 7.2% and 11.4%; FSH, 8.0% and 13.2%; and PRL, 8.1% and 11.4%. We performed the GnRH RIA as described by Mongiat et al. (2006); assay sensitivity was 1.5 pg, and intraassay and inter-assay coefficients of variation were 7.1% and 11.6%, respectively.

### Statistical analysis

We analyzed hormonal levels in PND13 and adult animals, pulsatility and puberty parameters, and estrous cycles by one-way or two-way analysis of variance (ANOVA) using Statistica, version 5, software (StatSoft Inc., Tulsa, OK, USA). We analyzed GnRH-induced LH in adults, IP_3_/inositol, Western blots, and LH and FSH released to culture media by repeated measures two-way ANOVA (Statistica, version 5). Data were transformed when the test for homogeneity of variances so required. Results are expressed as mean ± SE, and *p* < 0.05 is considered significant.

## Results

### Effects of neonatal BPA exposure on basal and GnRH-stimulated gonadotropin secretion and on GnRH pulsatility in infantile female rats

**Figure 1:**
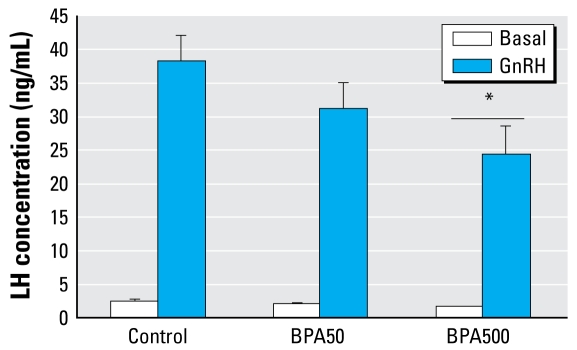
*In vivo* GnRH-induced LH release in PND13 rats. Values shown are mean ± SE serum LH in basal (saline) and GnRH-stimulated conditions (after 15 min) in control (*n* = 11), BPA50 (*n* = 9), and BPA500 (*n* = 11) treatment groups. Significance determined by two-way ANOVA: interaction, not significant; main effect chronic treatment, ^*^p < 0.05, compared with the corresponding control group.

On PND13, BPA500-treated animals had significantly lower basal serum LH than did controls [mean ± SE: control, 2.44 ± 0.28 ng/mL (n = 11); BPA50, 1.89 ± 0.18 ng/mL (n = 9); BPA500, 1.57 ± 0.18 ng/mL (n = 11); control vs. BPA500, p < 0.05]. GnRH significantly increased serum LH in all groups, but exposure to BPA dose dependently lowered GnRH-induced LH release, reaching statistical significance at the higher dose (Figure 1). However, the stimulated to basal ratio was not different among groups [GnRH-stimulated LH (fold increase), mean ± SE: control, 18.10 ± 3.01; BPA50, 17.00 ± 1.80; BPA500, 16.23 ± 2.50; not significant]. We observed no differences in either basal or GnRH-stimulated FSH levels (data not shown).

Next, we evaluated the response to GnRH in pituitary cells from control and high-dose animals cultured in vitro, a situation in which cells were deprived from the regulatory inputs from the gonads and the brain. Pituitary cells from the BPA500 group released significantly less LH to the media than controls, basally and after GnRH (1.10^−9^ M and 1.10^−7^ M) stimulation, similar to the in vivo results. For 1.10^−7^ M GnRH, LH concentrations were as follows: for control, 17.37 ± 1.66 ng/50,000 cells for basal and 46.75 ± 7.81 ng/50,000 cells for GnRH; for BPA500, 7.67 ± 0.87 ng/50,000 cells for basal and 24.43 ± 4.08 ng/50,000 cells for GnRH (n = 7; control vs. BPA500, p < 0.05). Again, percent increases were similar between groups for LH; after 1.10^−7^ M GnRH, percent increases were 277.4 ± 56.3% for control and 334.3 ± 68.0% for BPA500 (not significant). We observed no differences in basal or GnRH-induced FSH levels between groups (data not shown).

**Figure 2:**
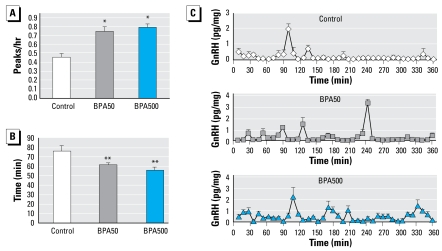
Effects of neonatal exposure to BPA on GnRH pulsatility from hypothalamic explants studied *ex vivo*. (*A*) Frequency of GnRH pulses (*n* = 4). (*B*) GnRH interpulse interval (*n* = 4). (*C*) Representative pulsatility patterns for the three BPA treatment groups. ^*^p< 0.01, and ^**^p < 0.02, by ANOVA.

In GnRH pulsatility studies, both BPA-treated groups exhibited significantly higher pulse frequency than controls, as well as an increased number of peaks per hour and a reduced interpulse interval (Figure 2A, B). Representative profiles of pulsatile GnRH release are shown in Figure 2C. We found no differences in the area under the concentration curve among treatments (data not shown).

Because we found differences in GnRH pulsatility with these two BPA doses, we carried out another experiment using hypothalami from animals injected as before but with a 10-fold lower dose of BPA (5 μg) (BPA5). Interestingly, we found that BPA5 animals also exhibited significantly higher pulse frequency than controls: 0.46 ± 0.04 peaks/hour for controls, and 0.83 ± 0.10 peaks/hr for BPA5 (*n* = 4; *p* < 0.05).

### Effects of neonatal BPA exposure on puberty onset, estrous cycles, pituitary function, and GnRH pulsatility and signaling in adulthood

**Figure 3:**
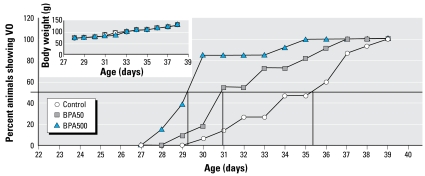
Percent of control, BPA50, and BPA500 animals showing VO. Inset: body weights of same animals (*n* = 11–15).

Early VO is a characteristic sign of advanced puberty. In rats treated with BPA50 and BPA500, VO occurred at earlier ages than in controls (Figure 3): control, 34.93 ± 0.71 days; BPA50, 32.46 ± 0.71 days; and BPA500, 30.15 ± 0.58 days (*p* < 0.05). We found no significant differences in body weight among the three groups (Figure 3).

In adulthood, only animals from the the BPA500 group showed irregular estrous cycles, with high prevalence of estrus after PND90, whereas proestrus and diestrus were markedly reduced. The percentage of days (± SE) in each stage of the cycle for the three groups are as follows: control: diestrus = 49.5 ± 0.5%, proestrus = 25.0 ± 0.8%, estrus = 25.5 ± 1.2%; BPA50: diestrus = 50.8 ± 5.1%, proestrus = 22.7 ± 3.2, estrus = 25.8 ± 2.7; BPA500: diestrus = 0.8 ± 0.8%, proestrus = 1.6 ± 0.9%, estrus = 97.7 ± 1.5%; (for BPA500 vs. control, *p* < 0.05).

**Figure 4:**
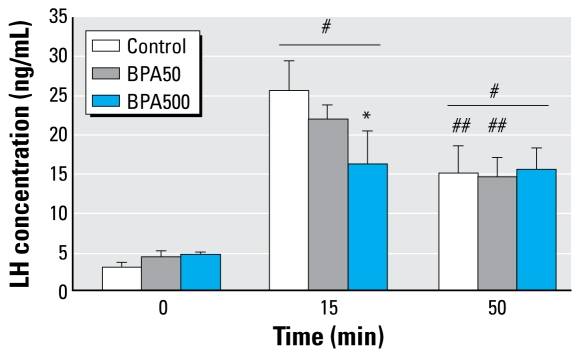
*In vivo* GnRH-induced LH release in adult rats in estrus. Values shown are mean ± SE serum LH in basal (saline) and GnRH-stimulated conditions (after 15 and 50 min) in control, BPA50, and BPA500 treatment groups (*n* = 7 per group). Significance determined by two-way repeated measures ANOVA: interaction *p* < 0.05. ^*^ *p* < 0.05 compared with control. ^#^*p* < 0.05 compared with 0 min. ^##^*p* < 0.05 compared with 15 min.

We also studied pituitary function in adulthood, both *in vivo* and *in vitro* and GnRH pulsatility *ex vivo*. For these studies we used control and BPA animals in estrus. We found no differences in serum gonadotropins on the morning of estrus. LH concentrations (mean ± SE) were 3.15 ± 0.65 ng/mL for control, 4.66 ± 0.41 ng/mL for BPA500, and 3.47 ± 0.47 ng/mL for BPA50; FSH concentrations were 5.67 ± 0.90 ng/mL for control, 6.18 ± 0.55 ng/mL for BPA500, and 6.98 ± 0.44 ng/mL for BPA50. As shown in Figure 4, 15 min after *in vivo* GnRH injection, serum LH increased in all groups; levels were lower in both BPA-treated groups, but this attained statistical significance only in high-dose animals. After 50 min of stimulation, LH was still increased, without differences among groups. Interestingly, in both the control and BPA50 groups at 50 min, LH levels were significantly lower than those at 15 min, whereas in the BPA500 group, LH levels did not vary between these time points.

Regarding GnRH pulsatility, adult animals neonatally exposed to either dose of BPA exhibited higher pulse frequency than controls, as demonstrated in PND13 females: control, 0.60 ± 0.05 peaks/hr; BPA50, 0.81 ± 0.03 peaks/hr; BPA500, 0.83 ± 0.04 peaks/hr (*n* = 7; control vs. BPA50 and BPA500, *p* < 0.05].

**Figure 5:**
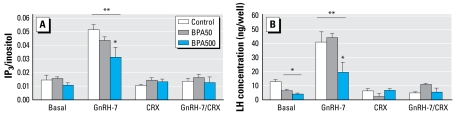
*In vitro* GnRH-induced IP_3_ formation (*A*) and LH release (*B*) in adult anterior pituitary cell cultures from control, BPA50, and BPA500 animals (*n* = 6 per group). (*A*) Effect of GnRH (1.10^−7^ M), CRX (1.10^−6^ M), and both combined on IP_3_ production after 30 min of stimulation; values are expressed as [^3^H]IP_3_ (cpm)/[^3^H]inositol incorporated by cells. (*B*) LH (ng/500,000 cells) secreted into media after 30 min incubation with these stimuli. Two-way repeated measures ANOVA: interaction: *p* < 0.05. ^*^p < 0.05 compared with control. ^**^p < 0.05 compared with basal in each group.

In addition, we determined GnRH responsiveness *in vitro* in primary pituitary cell cultures. We analyzed LH secretion and GnRH-stimulated second messengers. Neonatal exposure to BPA dose dependently impaired GnRH-induced IP_3_/inositol compared with controls (Figure 5A). Cells from the BPA-treated groups released less basal LH than controls; BPA500 also released less LH in response to GnRH (Figure 5B). In all cases, CRX abolished GnRH-stimulated IP_3_/inositol increase and LH secretion. IP_3_ formation and medium LH were highly correlated (*r*= 0.9641; *p* < 0.005).

**Figure 6:**
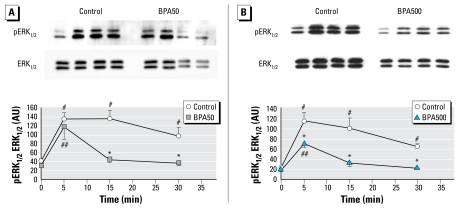
GnRH (1.10^−7^ M)-induced activation of ERK_1/2_ [arbitrary units (AU)] in anterior pituitary cell cultures from adult control, BPA50, and BPA500 animals. (*A*) Representative Western blot of control and BPA50 pituitary cells (top) and quantification (bottom). *n* = 4 per group. (*B*) Representative Western blot of control and BPA500 pituitary cells (top) and quantification (bottom). For (*A*), two-way repeated measures ANOVA, interaction: *p* < 0.05, and ^*^p < 0.05 compared with control. ^#^*p* ≤ 0.05 compared with 0-min control. ^##^*p* ≤ 0.05 compared with 0-min BPA50. For (*B*), two-way repeated measures ANOVA, interaction: *p* < 0.05. ^*^p ≤ 0.05 compared with control. ^#^*p* ≤ 0.05 compared with 0-min control. ^##^*p* ≤ 0.05 compared with 0-min BPA500.

Finally, we analyzed the GnRH-activated ERK_1/2_ pathway in these experimental groups (Figure 6A, B). In controls, ERK_1/2_ phosphorylation was rapid (apparent after 5 min of GnRH stimulation) and sustained (still significantly activated after 30 min), as classically reported (Liu et al. 2002; Sundaresan et al. 1996). In contrast, both BPA groups exhibited a rapid but transient activation of ERK_1/2_. In addition, the highest levels reached in the BPA500 group were significantly lower than levels in controls after 5 min stimulation. The BPA50 group reached the same levels as controls after 5 min stimulation, but a quick significant decrease to basal levels occurred thereafter.

### Effects of neonatal exposure to BPA on PRL levels in infantile and adult rats

We observed no significant differences in serum PRL between groups on PND13 (data not shown). Nevertheless, serum PRL was significantly higher in BPA500 adult animals than in controls, and levels in BPA50 animals were similar to those in controls [control, 4.34 ± 0.67 ng/mL (*n* = 8); BPA50, 5.42 ± 0.66 ng/mL (*n* = 7); BPA500, 11.34 ± 1.31 ng/mL (*n* = 8); BPA500 vs. control, *p* < 0.05].

## Discussion

Here we report significant effects of neonatal exposure to BPA on the hypothalamic–pituitary–gonadal axis of infantile and adult female Sprague-Dawley rats. In 13-day-old animals, an age when the pituitary is highly sensitive to releasing and inhibiting stimuli, neonatal exposure to BPA decreased basal and GnRH-stimulated serum LH, and this reached statistical significance at the higher dose (BPA500). When tested *in vitro*, BPA500-treated animals also released less LH, basally and after GnRH-stimulation. Interestingly, BPA-induced alterations in GnRH pulsatility; the three doses of BPA tested in this case (BPA5, BPA50, and BPA500) increased GnRH pulse frequency, with an increase in the number of peaks per hour. Similar effects on GnRH pulsatility have been demonstrated by estradiol and DDT administration (Matagne et al. 2004; Rasier et al. 2007). The decrease in basal and GnRH-induced LH release by BPA may be the consequence of the increase in GnRH pulse frequency, leading to desensitization of the pituitary, as also suggested by others (Knobil and Hotchkiss 1988), although we cannot exclude some direct effect of BPA on LH secretion.

Neonatal treatment with high and low doses of BPA also affected puberty onset, dose-dependently advancing VO, without affecting body weight. A similar advance in puberty onset has also been described after a single injection of estradiol on PND10 (Matagne et al. 2004) or after 5 days of treatment (PND6–PND10) (Rasier et al. 2007). The insecticide DDT, another endocrine disruptor, has also been reported to advance puberty onset (Rasier et al. 2007). Our results show that even though Sprague-Dawley rats have been described as possessing a low sensitivity to estrogenic compounds (Steinmetz et al. 1997), the effect of BPA, administered during a period critical for development, is similar to that described after early exposure of females to estradiol. Other groups also found early VO in animals treated with BPA perinatally (from pregnancy through lactation) (Durando et al. 2007) or postnatally (Kato et al. 2003), this last group using doses twice as high as those used in the present study.

Puberty changes occur as a consequence of the activation of the hypothalamic–pituitary–gonadal axis (Buck Louis et al. 2008). As discussed above, BPA, as well as estradiol and DDT, when administered to immature rats, induced acceleration in GnRH pulsatility (Rasier et al. 2007). In addition, there is physiologically a developmental reduction in serum LH before puberty onset (Rasier et al. 2007), so the decreased LH levels described above could result from either negative feedback, accelerated maturation, or both. Therefore, here, we show for the first time that BPA could be causing precocious hypothalamic–pituitary maturation and thus inducing precocious puberty.

In adults the higher dose significantly disrupted estrous cycles with a high prevalence of estrus. Although we found no effect on estrous cycles with the lower dose (BPA50), we cannot exclude the possibility of loss of cyclicity later in life. Durando et al. (2007) and Kato et al. (2003) also reported altered estrous cycles, the latter with irregular cycles after a single BPA dose of 1 mg/animal and persistent estrus after a single dose of 4 mg/animal (a dose 8 times higher than our BPA500 dose). Similar results have also been reported with other xenoestrogens, such as the phytoestrogen coumestrol (Kouki et al. 2005) and DDT (Rasier et al. 2007).

In the present study, neonatal treatment with BPA did not modify basal serum gonadotropins but did have an impact on the *in vivo* GnRH-induced LH release in adult females, with BPA500 reducing the response to the decapeptide; to our knowledge, this is the first report of such an effect. In addition, we also observed accelerated GnRH pulse frequency in adult females neonatally exposed to high and low BPA, demonstrating that the effect found in infantile animals persists throughout life. Moreover, adult pituitary cells from animals neonatally exposed to BPA showed decreased basal LH release, but only the pituitaries from high-dose animals released less LH after GnRH stimulation. When analyzing signal transduction pathways elicited by GnRH, we found that in pituitary cells from BPA-treated animals, GnRH-stimulated IP_3_ formation was impaired, showing dose dependency for this effect and tightly correlating with LH secretion. This is in agreement with the concept that the IP_3_–calcium pathway is involved in gonadotropin release. In contrast, we found that the pattern of ERK_1/2_ activation after GnRH stimulation was altered in cultures from both BPA treatment groups. Controls showed the classical rapid and sustained activation described in gonadotropes (Sundaresan et al. 1996), whereas both BPA treatment groups showed a peak at 5 min and a subsequent pronounced inactivation. The ERK_1/2_ pathway links signals from the cell surface to the cytoplasm and nucleus; this results in transcriptional and mitogenic events. In the case of GnRH-induced ERK activation, this pathway is involved in LHβ (Liu et al. 2002) and GnRH receptor expression (Lin and Conn 1999) and probably mediates the mitogenic actions of GnRH on gonadotropes (Lewy et al. 2003), among other actions. High-dose animals showed a lower maximal ERK_1/2_ phophorylation than the other groups; this is in line with the results obtained with IP_3_, because activation of ERK_1/2_ is downstream to PKC activation (Chamson-Reig et al. 1999; Lewy et al. 2003; Sundaresan et al. 1996). These results show for the first time different intracellular responses to GnRH in pituitaries from BPA-treated animals.

We found no differences in serum PRL titers in infantile animals after BPA treatment. In contrast, BPA500 animals showed hyperprolactinemia. These results agree with a previous study showing hyperprolactinemia at PND20 and PND30 but not PND15 (Khurana et al. 2000). Other studies have shown hyperprolactinemia at PND30 in males treated prenatally with BPA (Ramos et al. 2003), whereas others reported an increase in serum PRL in adult females ovariectomized and treated with BPA (Goloubkova et al. 2000).

Taken together, these results demonstrate that exposure to high and low doses of BPA, an environmental endocrine disruptor, in a period of time critical for development alters reproductive parameters in infantile and adult animals, causing precocious hypothalamic–pituitary maturation and precocious puberty, altering GnRH pulsatility in infantiles and adults, and severely affecting GnRH signaling in the adult pituitary. More studies are needed, including those with additional doses of BPA, to further understand the mechanisms underlying these effects.
